# Alisol B 23-Acetate Ameliorates Azoxymethane/Dextran Sodium Sulfate-Induced Male Murine Colitis-Associated Colorectal Cancer *via* Modulating the Composition of Gut Microbiota and Improving Intestinal Barrier

**DOI:** 10.3389/fcimb.2021.640225

**Published:** 2021-04-29

**Authors:** Huai-Chang Zhu, Xiao-Kang Jia, Yong Fan, Shao-Hua Xu, Xiao-Yan Li, Ming-Qing Huang, Meng-Liu Lan, Wen Xu, Shui-Sheng Wu

**Affiliations:** ^1^ College of Pharmacy, Fujian University of Traditional Chinese Medicine, Fuzhou, China; ^2^ Centre of Biomedical Research & Development, Fujian University of Traditional Chinese Medicine, Fuzhou, China; ^3^ Academy of Integrative Medicine, Fujian University of Traditional Chinese Medicine, Fuzhou, China

**Keywords:** Alisol B 23-acetate, colitis-associated cancer, gut microbiota, intestinal barrier, TLR/NF-κB/MAPK

## Abstract

Hunting for natural compounds that can modulate the structure of the intestinal flora is a new hotspot for colitis‐associated cancer (CAC) prevention or treatment. Alisol B 23-acetate (AB23A) is a natural tetracyclic triterpenoid found in *Alismatis rhizoma* which is well known for dietary herb. *Alismatis rhizoma* is often used clinically to treat gastrointestinal diseases in China. In this study, we investigated the potential prevention of AB23A in male mouse models of azoxymethane (AOM) and dextran sulfate sodium (DSS)-induced CAC. AB23A intervention alleviated the body weight loss, disease activity index, colon tumor load, tissue injury, and inflammatory cytokine changes in CAC mice. AB23A intervention leads to remarkable reductions in the activation of TLR, NF-κB and MAPK. AB23A significantly decreased the phosphorylation of p38, ERK, and JNK and up-regulated mucin-2 and the expression of tight junction proteins. The gut microbiota of AB23A-interfered mice was characterized with high microbial diversity, the reduced expansion of pathogenic bacteria, such as *Klebsiella*, *Citrobacter*, and *Akkermansia*, and the increased growth of bacteria including *Bacteroides*, *Lactobacillus*, and *Alloprevotella*. These data reveal that AB23A has the potential to be used to treat CAC in the future.

## Introduction

Colitis-associated cancer (CAC), the fourth leading reason of tumor-associated death all over the world, is one of the major colorectal cancer types (CRC) (Huyghe et al., 2019). Inflammation is a known risk factor for the development of CAC (Grivennikov, 2013; Zhu et al., 2019). The most popular treatments for CAC are surgery and chemotherapy. Chemotherapeutic medications, such as 5-fluorouracil, are usually used to treat patients with advanced stages of CAC, either alone or in conjunction with an adjuvant, such as oxaliplatin or Avastin (Siegel et al., 2014; Van Cutsem et al., 2015; Colombo et al., 2016; Miller et al., 2019). However, although current treatment methods are effective in improving CAC and survival, side effects are still very troublesome, such as weight loss, nausea, vomiting, and the risk of infection complications caused by weakened immunity (Sanders et al., 2016; Kamarudin et al., 2019). Therefore, it is essential to study effective and safe anti-CAC treatment methods. Natural compounds from Traditional Chinese medicine (TCM) have been reported to prevent and treat CAC with few side effects (Sanders et al., 2016).Thus, finding new and effective ingredients against CAC from natural compounds is of great significance.

Alisol B 23-acetate (AB23A), the main ingredient in *Alismatis rhizoma*, has been reported to present excellent bioactivity, including anti-inflammatory (Jiang et al., 2006), anti-microbial (Jin et al., 2012), and anti-proliferative properties (Law et al., 2010; Zhao et al., 2017). Chinese common dietary herb preparations (LiuweiDihuangWan, Zexie decoction) mainly containing *Alismatis rhizoma* are clinically used for gastrointestinal and inflammation diseases including obesity and diabetes (He, 2007; Zhou W, 2002; Xu et al., 2014). A previous pharmacokinetic study showed that the bioavailability of AB23A is poor and excreted in the form of feces (Xu et al., 2017). Modern pharmacological studies have shown that AB23A can exhibit good anti-tumor properties *via* influencing the PI3K/Akt/mTOR pathway, ROS generation, and JNK activation (Wang et al., 2017; Zhao et al., 2017; Wang et al., 2018). However, the pharmacological mechanism of the relationship between AB23A and CAC remains unclear.

Developments in microbiome technology have provided numerous clinical and scientific research studies showing that CAC is closely related to gut microbiota (Kang and Martin, 2017). Many current studies have shown that the intestinal flora plays an important role in the formation of the inflammatory microenvironment and the promotion of tumor growth (Brennan and Garrett, 2016). The gut microbiota of CAC mice is characterized by reduced microbial diversity and reduced abundance of probiotics (including Bacteroides, *Lactobacillus*) (Zhao and Jiang, 2020), and *Alloprevotella* (Wei et al., 2018), and the expansion of pathogenic bacteria, such as *Proteobacteria* (Shin et al., 2015; Litvak et al., 2017), Muribaculaceae (Pereira et al., 2020), *Akkermansia* (Ijssennagger et al., 2015), *Klebsiella* (Olm et al., 2019), and *Citrobacter* (Collins et al., 2014). Gut microbiome dysbiosis leads to changes in cytokine activity, inflammatory signaling pathway activation (e.g., TLR4, TLR5, NF-κB, and MAPKs), and intestinal barrier function weakening, all of which provide a pro-inflammatory microenvironment for tumor promotion (Fukata et al., 2009; Choi et al., 2013; Waldner and Neurath, 2015). Therefore, maintaining the steady state of the gut microbiota may be a promising strategy for the treatment and prevention of CAC. Natural compounds play an important role in restoring intestinal homeostasis (Zhang et al., 2012; Huang et al., 2019; Zeng et al., 2020). Thus, hunting for natural compounds modulating the structure of the intestinal flora is a new hotspot for CAC prevention or treatment. Here, we aimed to investigated AB23A whether produces many beneficial changes on mouse CAC model, and the possible mechanisms involved in the effect of AB23A prevention against CAC.

## Materials and Methods

### Materials

Azoxymethane and Dextran Sodium Sulfate were purchased from MP Biomedicals (Santa Ana, CA, USA). AB23A was purchased from Must Bio-Technology Co., Ltd (Chengdu, Sichuan, China.) ELISA kits were purchased from ABclonal Biotechnology (Wuhan, Hubei, USA). Protein lysis buffer was purchased from Beyotime (Shanghai, China). PVDF membrane was purchased from Millipore (Boston, MA, USA). ECL reagent kit was purchased from Thermo Fisher Scientific (USA). The information of all antibodies used in this study were shown in **Supplementary Table 1**.

### Animals

The Animal Ethics Committee of Fujian Medical University inspected and approved the animal experiments. (No: FJMU IACUC 2019-0066) and the operations on mice were carried out in compliance with Fujian Medical University’s ethical methods and programs for the use of laboratory animals. Shanghai Slake Laboratory Animal Co., Ltd. provided the mice (C57BL/6J male mice, 18-20 g) that needed in this study and raised in accordance with the ethical methods and programs of Fujian Medical University on the use of laboratory animals. Great attempts were made to keep animal cruelty to a minimum.

### Animal Experiment Operation

All experimental mice were placed in an SPF-level animal barrier. During animal experiments, the temperature in the animal laboratory was 22 ± 2°C, and the light and dark conversion cycle was 12 hours. After one week of free breeding and adaptation to the animal barrier environment, 30 mice were divided into three classes at random: a control group (*n* =10), an AOM/DSS-induced model (DSS) group (*n* =10), and an AB23A treatment group (*n* =10).

**Figure 1:**
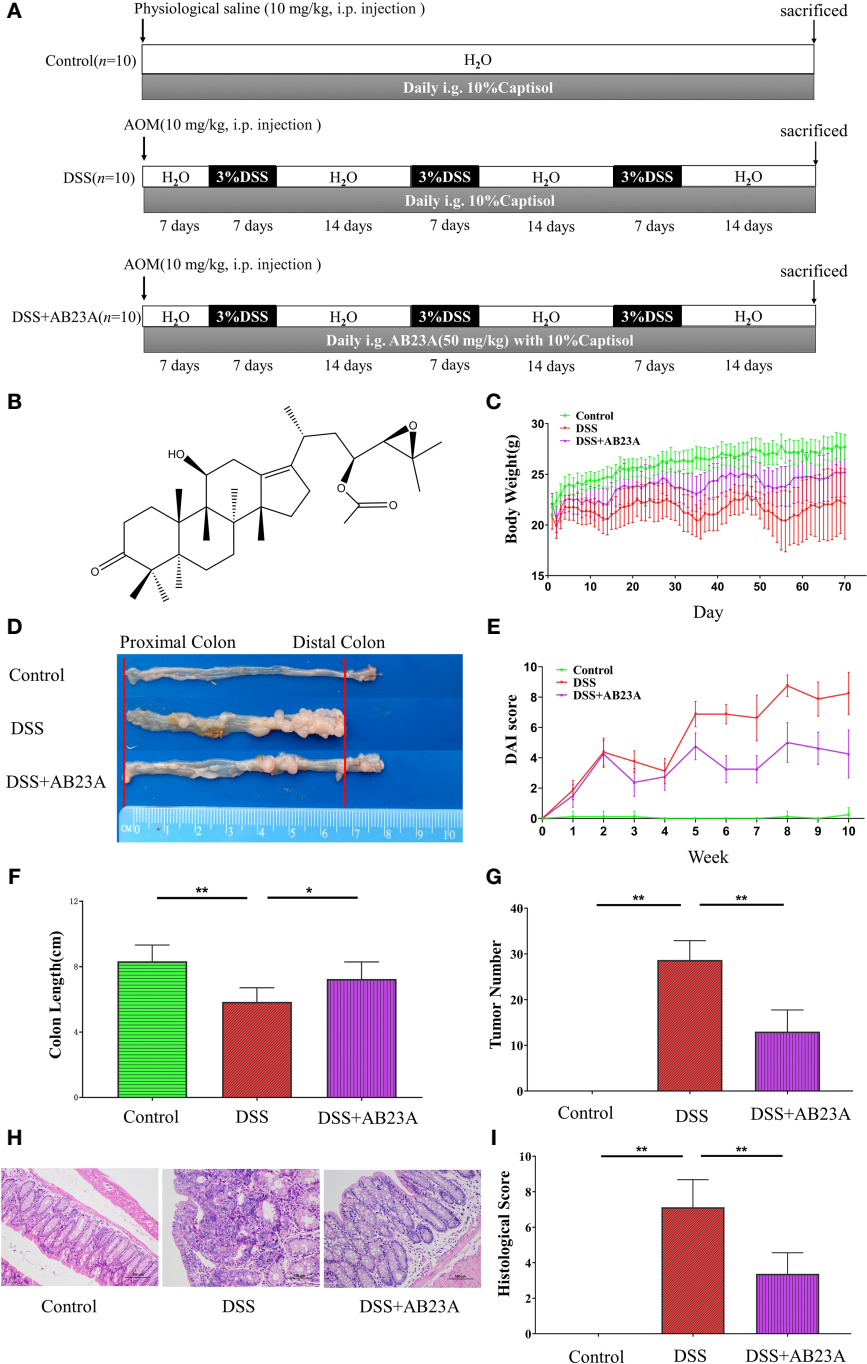
AB23A treatment ameliorated pathological damage in AOM/DSS-induced CAC mice. **(A)** The overview of the animal experiment design. **(B)** Chemical structure of AB23A. **(C)** Body weight changes in each group (n = 10). **(D)** Colon tumor burden in each group. **(E)** DAI scores (n = 10). **(F)** Colon lengths in each group (n = 6). **(G)** Colon tumor numbers in each group (n = 6). **(H)** Micrographs of HE-stained colon tissues in each group (n = 3). **(I)** Histological scores of each group (n = 3). Data are expressed as mean ± SD. Differences in **(B, D–F, H)** were analyzed by one-way ANOVA followed by Tukey’s post hoc test (*P < 0.05, **P < 0.01).

At the beginning of the experiment, mice in the DSS and AB23A groups received an intraperitoneal injection of AOM (10 mg/kg); mice in the normal group were intraperitoneally injected with physiological saline (10 mL/kg). On the seventh day, mice in the DSS and AB23A groups were given water containing 3% (w/v) DSS. After 7 days, normal sterile drinking water was provided for 14 days. The control group was given sterile drinking water throughout this procedure. This cycle was repeated thrice until mature tumors developed. The entire experiment period was 70 days. The AB23A group began intragastric administration of AB23A (50 mg/kg) and solvent (10% Captisol) daily on the first day of the experiment and the other two groups were given a corresponding dose of solvent (10% Captisol) daily (Meng et al., 2015; Meng et al., 2017). An overview of the animal experiment design is provided in the **Figure 1A**. Over the course of the experiment, mouse weights, stool shapes, and disease status were noted every day. At the end of the experiment, the mice were euthanized for sample collection.

For biochemical analysis, blood samples were centrifuged at 4000 g for 10 minutes at 4°C, transferred to EP tubes, and processed at 80°C. for biochemical analysis. The colon was carefully cut longitudinally to collect its contents. The colon was quickly washed with pre-chilled saline, and the number and location of tumors were recorded. One part of the pre-chilled colon tissue was fixed in 10% anhydrous formalin for HE, and another part was stored at −80°C for subsequent biochemical determination. During the experiment, all experimental tissue samples are performed on ice for as long as possible during operation.

### Assessment of CAC Mice

The disease activity index (DAI), which is commonly used to evaluate the progress of CAC, was calculated according to the weight change of the mouse (such as whether it has increased or decreased), the external characteristics of the stool, and the blood in the stool. The range of DAI is 0 ~ 4. The detailed evaluation criteria are provided in **Supplementary Table 2**.

The colon is placed on a blue background plate and the length will be measured on ice in time. The tissue was then fixed in tissue fixation fluid (10%) for one day for histological examination *via* staining with hematoxylin–eosin (HE). The histological scores ranged from 0 to 10 according to the extent of colon injury. The total scoring system was based on four parameters, namely, mucosal epithelium, cell infiltrate and edema, integrity of crypts, and goblet cell depletion. The detailed evaluation criteria are provided in **Supplementary Table 3**.

### Hematoxylin–Eosin Staining

Colon tissues that have been fixed in formaldehyde solution (10%) for over 24 h were washed thrice with PBS and then placed in ethanol for gradient dehydration for 1 h each time. Subsequently, the colon tissue was obtained, placed in xylene I and II solutions for transparent treatment, and then allowed to stand for 1 h. Afterward, paraffin embedding was carried out at 56-58 °C. The samples were sliced into sections after cooling at room temperature, placed in warm water for unfolding, mounted on glass slides, and then dried. The sections were soaked once more in xylene I and II solutions for dewaxing treatment, and immersed in 100%, 95%, 85%, and 75% ethanol for 1 min each time for rehydration. The parts were stained for 5 minutes with hematoxylin, then distinguished for 5-10 seconds with a 1 percent HCl solution, washed twice with water, and stained for 2 minutes with eosin. Ethanol dehydration treatment was carried out once more, followed by xylene I and II soaking for 3 and 5 min, respectively. Finally, the film was sealed with neutral gum and observed under a microscope.

### ELISA Kits

The relative expression of inflammatory cytokines, including IL-6, TNF-α, IFN-γ, and IL-10, in serum and colon tissue was measured with the ELISA kit (ABclonal Biotechnology Co., Ltd., USA). Ice-cold normal saline and colon tissue were mixed at a ratio of 1:10 (w/v) and homogenized, then centrifuged at 5000 g for 15 minutes, and the supernatant was taken to measure inflammatory cytokines.

### Western Blot Analysis

The purposed protein from the colon tissue were extracted *via* using tissue lysis buffer and denatured with sample loading buffer (5×) at 100°C. Total proteins were denatured and electrophoresed in 10% SDS-PAGE before being moved to the PVDF membrane. The membrane was blocked with blocking buffer for 2 hours at normal room temperature. and then incubated overnight on ice with the primary antibody (dilution, 1:1000). Thereafter, wash the membrane with TBST, 3 times for 10 minutes each time. Then incubation with the corresponding secondary antibody (dilution: 0.5:5000) was carried out for 2 hours at normal room temperature. The ECL reagent kit was used to detect the expression of the protein in the bands, and Image J software was used to show striped images (NIH, USA) and detected the luminous intensity of the bands. β-Actin was used to present the standard of same loading of protein.

### Sequencing of Feces of CAC Mice

Fecal samples of mice were collected on the 70th day and used for gut microbiota sequencing. Anal stimulation was used to stimulate mice to defecate. When the mouse’s defecation is about to end, the stool is immediately clamped with sterile forceps and placed in a sterile tube, and then immediately stored in a liquid nitrogen tube. The collected feces are finally stored in a refrigerator at -80°C. The statement in detail of our sequencing of feces of CAC mice can be find in **Supplementary Material**.

### Bioinformatic Analysis of Sequencing Data

Bioinformatics analysis was performed through NovoMagic, a data analysis platform independently developed by Novogene Co., Ltd. Analysis tools, algorithms, codes, and data are visible in the **Supplementary Material**.

### Statistical Analysis

All data were expressed as mean ± SD. Multiple comparisons were performed using one-way ANOVA. When *P* < 0.05 or *P* < 0.01, the data were considered statistically significant. The experimental data calculation and analysis were operated *via* GraphPad Prism software (version 8.3.0).

## RESULTS

### AB23A Ameliorated Pathological Damage in AOM/DSS-Induced CAC Mice

**Figure 1** shows that AB23A administration significantly ameliorates AOM/DSS-induced CAC, as evidenced by marked reductions in weight loss (**Figure 1C**), decreased tumor burden (**Figure 1D**), significantly lower DAI scores (**Figure 1E**), and relief of colonic shortening (**Figure 1F**). The incidence of dysplasia in each group was: 0% in the normal group; 100% in the DSS group and 100% in the AB23A group. Although the dysplasia rate of the AB23A intervention group was not improved compared with the DSS group, the size and number of colon tumors in mice were reduced. The tumor burden of the DSS group was significantly more severe than that of the AB23A group. Compared with the control group (0.00 ± 0.00), the DSS group showed a significant increase in colon tumor number (28.67 ± 4.23) whereas the AB23A group demonstrated a significant reduction in tumor number (13 ± 4.73; **Figure 1G**). These results indicate that the CAC model had been successfully constructed and illustrate the primary macroscopic protection effect of AB23A. Compared with those of the control group, the colon tissue sections of the DSS model group showed very obvious pathological changes, including the destruction of colonic epithelial cells, distortion of crypts, the loss of goblet cells, colon epithelial damage, and varying inflammatory infiltration (**Figure 1H**). By contrast, the AB23A group revealed a marked reduction of these symptoms. The histological scores can directly reflect the severity of colon pathological damage (**Figure 1I**). The histological scores of the DSS group were clearly much higher than those of the control and treatment groups. This result indicates that intervention with AB23A alleviates the clinical signs and tumor development of AOM/DSS-induced CAC.

### AB23A Attenuated the Production of Inflammation-Related Cytokines of AOM/DSS-Induced CAC Mice

**Figure 2:**
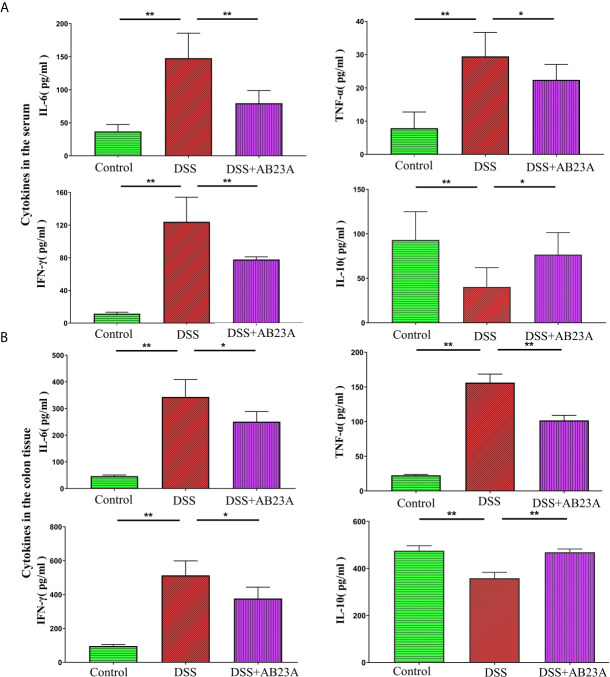
Effects of AB23A on serum levels of cytokines in serum and colon tissue. **(A)** The expression of IL-6, TNF-α, IL-10, and IFN-γ in serum. **(B)** The expression of IL-6, TNF-α, IL-10, and IFN-γ in colon tissue. Data are expressed as mean ± SD (n = 8 in the serum and n = 4 in colon tissue). Differences were analyzed by one-way ANOVA followed by Tukey’s post hoc test (*P < 0.05, **P < 0.01).

A wide variety of research shows that chronic inflammation is one of the primary risk factors for initiation, proliferation, or metastasis of cancer. Considering the inter-triggering mechanism of cancer progression and inflammation, we assessed the production of pro-inflammatory cytokines, including IL-6, TNF-α, and IFN-γ, and the anti-inflammatory cytokine IL-10 in CAC mice in colon tissue and serum. In the serum, compared with those in the DSS group, the pro-inflammatory cytokines in the AB23A group manifested significantly decreasing trends, especially IL-6 (*P* < 0.01), TNF-α (*P* < 0.05), and IFN-γ (*P* < 0.01). AB23A intervention significantly increased the expression of IL-10 (*P* < 0.05; **Figure 2A**). In colon tissue (**Figure 2B**), pro-inflammatory cytokines in AB23A group, such as IL-6 (*P* < 0.05), TNF-α (*P* < 0.01), and IFN-γ (*P* < 0.05) significantly decreased compared with the DSS group, while the expression of IL-10 (*P* < 0.01) was suppressed. Thus, AB23A markedly alters the cytokine profile of AOM/DSS-induced CAC mice by suppressing the expression of proinflammatory-related cytokines and upregulating the expression of anti-inflammatory cytokines to relieve gut inflammation, which is considered an inducing factor of colon cancer.

### AB23A Drove the Composition of Gut Microbiota in CAC Mice Toward Normal Level

**Figure 3:**
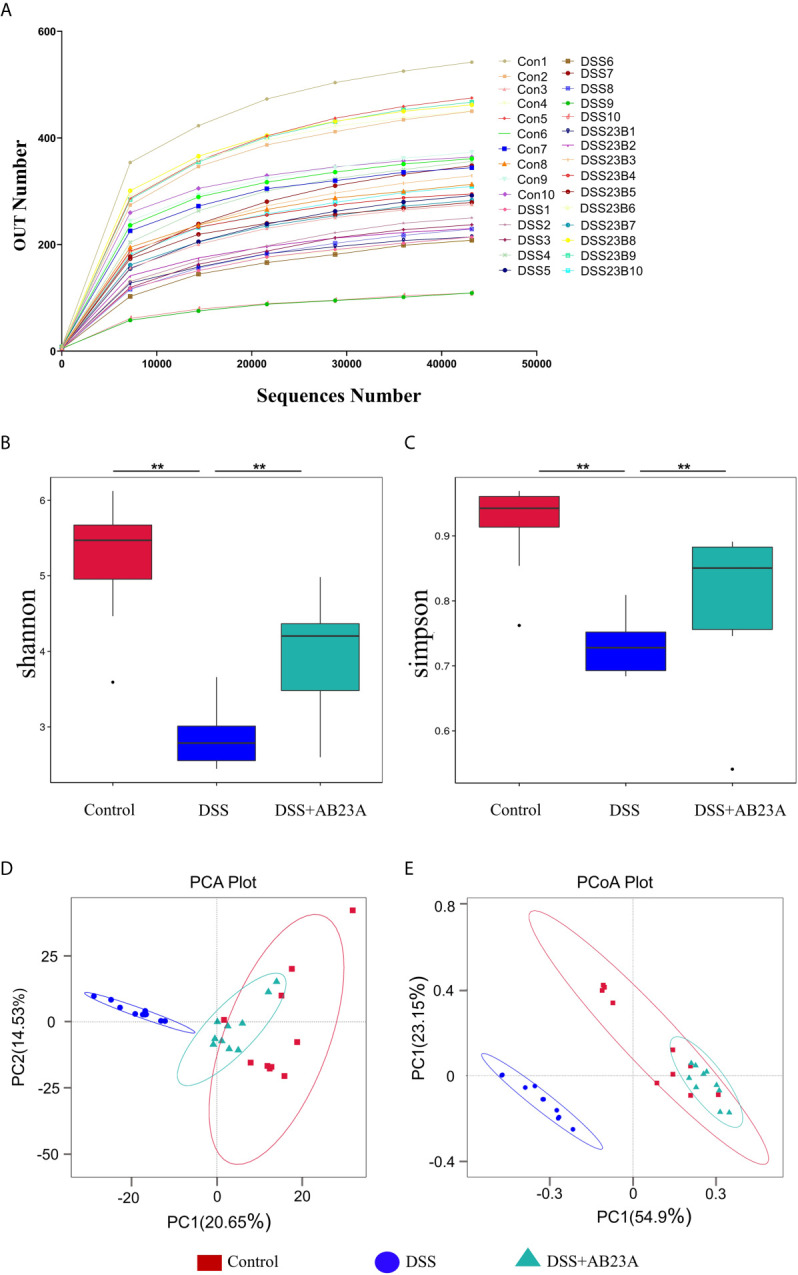
Richness and diversity of fecal microbiotas among groups. **(A)** The rarefaction curve was used to measure sample rationality. **(B, C)** α-Diversity analysis: Shannon and Simpson box plots. **(D)** PCA of gut microbiota. **(E)** PCoA of gut microbiota (n = 10 samples per group. (vs. DSS: **P < 0.01).

Alterations in gut microbiota were found by the sequencing of the 16s rRNA genes extracted from the control, DSS, and AB23A groups. According to the sequencing results, 89,936 tags were detected in each sample on average. Through the evaluation of the quality of tags, the effective data for each sample is 86,363 tags. Finally, the effective ratio of quality control reached 66.68%. The obtained sequence was clustered into an OTU unit with 97% identity, and finally divided into 1113 OTU units. The OTU sequences were annotated to species. We found that the ends of the dilution curves of all samples tended to be flat (**Figure 3A**), thus indicating that the amount of our sequencing data is reasonable, that the distribution of species richness and uniformity in the sample is relatively concentrated, and that no particularly discrete sample is present.

α-Diversity indices, including Chao1, Shannon, Observed_species, Simpson, and ACE, are commonly used to for evaluating the richness and diversity of microbiota. The Simpson index has three display forms, namely Simpson’s Index (D), Simpson’s Index of Diversity (1-D), and Simpson’s Reciprocal Index (1/D), they have similar effects on reflecting community diversity but the calculated results are in different forms. We used the Simpson’s Index of Diversity (1-D) display format which reflected the trend that is consistent with the Shannon index. As shown in **Figures 3B, C**, the diversity indices, including the Shannon and Simpson indices, manifested similar tendencies. The DSS group had the lowest other α-diversity indices of the three groups, and the other two groups had similar values. The other α-diversity indices were presented in the supplementary material (**Supplementary Table 4**). This result indicates significant microbiota destruction in the DSS group. In summary, AB23A intervention can obviously improve the abundance and diversity of the gut microbiota.

β-Diversity analysis was used to assess the variance of diversity among different groups. We performed β-diversity analysis to generate principal component analysis (PCA) and PCoA plots. As shown in **Figures 3D, E**, PCA and PCoA yielded clearer clusters in the control and AB23A groups than in the DSS group. β-Diversity analysis revealed Species distribution in the AB23A group tended to be closer to the control group.

**Figure 4:**
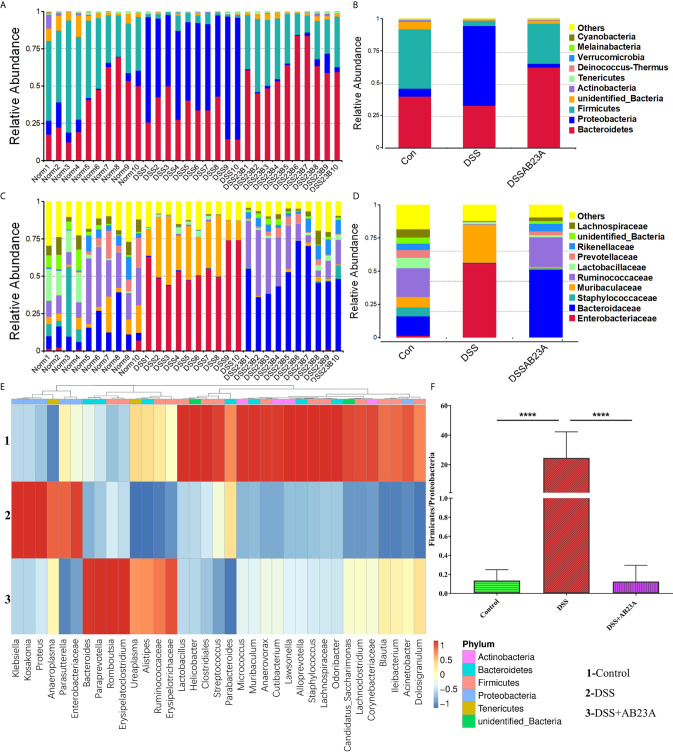
Effect of AB23A on the composition of gut microbiota. **(A, B)** Top 10 species of gut microbiota at the phylum level. **(C, D)** Top 10 species of gut microbiota at the family level. **(E)** Heat map of 35 species of gut microbiota at the genus level. **(F)** Ratio of Proteobacteria to Firmicutes (vs. DSS: ****P < 0.0001).

To determine which bacteria are altered by AB23A and, in turn, affect disease progression in CAC, we analyzed the top 10 species with the highest abundance at different taxonomic levels. Compared with that in the control group, Proteobacteria increased whereas Firmicutes decreased at the phylum level in the DSS group (**Figures 4A, B**). At the family level, the proportions of Enterobacteriaceae, *Akkermansiaceae*, and Muribaculaceae increased whereas the proportions of *Prevotellaceae*, *Rikenellaceae*, Ruminococcaceae, and Bacteroidaceae decreased in the DSS group compared with those in the control group (**Figures 4C, D**). The proportions of these families in the AB23A group were roughly similar to those in the control group. We selected the top 35 genera with the highest abundance (**Figure 4E**) for in-depth analysis of differences among groups. The abundance and diversity of these genera in the DSS group clearly decreased. Compared with the control and AB23A groups, the DSS group showed higher proportions of *Klebsiella*, *Citrobacter*, and *Akkermansia* and lower proportions of Bacteroides, *Alloprevotella*, and *Lactobacillus*. The DSS administration increased the ratio of Proteobacteria to Firmicutes sharply (**Figure 4F**). AB23A intervention reversed this increase and restored the ratio of Proteobacteria to Firmicutes to levels similar to that in the control group.

**Figure 5:**
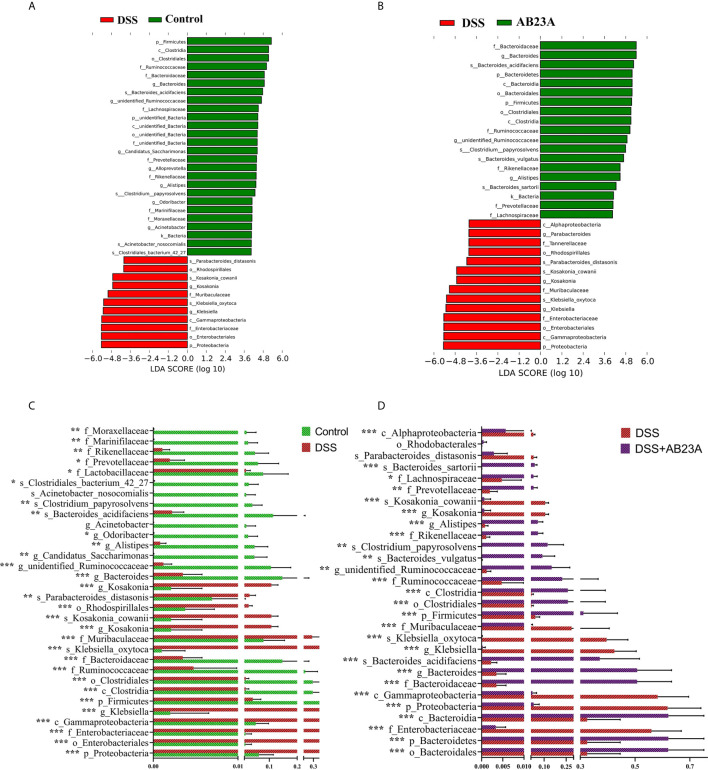
Species distributions of gut microbiota in each group. **(A)** LDA value-based distribution histogram between the control group and the DSS group (LDA > 4.0). **(B)** LDA value-based distribution histogram between the DSS group and the AB23A group (LDA > 4.0). **(C)** Comparison of relative abundance of different bacteria between the control group and the DSS group. (D) Comparison of relative abundance of different bacteria between the DSS group and the AB23A group. Differences were analyzed by Unpaired T-test (*P < 0.05, **P < 0.01, ***P < 0.001, n = 10).

In order to find the differential flora regulated by AB23A, LEfSe was performed and investigate the influences of AB23A on the gut microbiota of CAC mice. LEfSe analysis indicated that Proteobacteria (class Gammaproteobacteria), Enterobacteriales (order and family levels), Enterobacteriaceae, Muribaculaceae, and *Klebsiella* (genus and species levels) are the main kinds of bacteria that contribute to gut microbiota dysbiosis in the CAC mice of DSS group compared with the control group. (**Figure 5A**). Between DSS and AB23A, the microbiota in the AB23A group displayed the enrichment of Bacteroidetes at the genus and family levels, Clostridia (class and order levels), Ruminococcaceae, *Bacteroides_acidifaciens* and unidentified_Ruminococcaceae (**Figure 5B**). The relative abundances of significantly altered taxa are visualized in **Figures 5C, D**. In comparison to the two other categories, DSS administration induced a large rise in certain pathogens in Proteobacteria (e.g., *Klebsiella*, *Citrobacter*, and Enterobacteriaceae). At the same time, some SCFA-related bacteria (e.g., *Rikenellaceae* and *Prevotellaceae)* and potential beneficial bacteria, such as *Bacteroides acidifaciens*, *Bacteroides vulgatus*, and *Lactococcus*, decreased.

### AB23A Significantly Suppressed the Activation of TLR4, TLR5, NF-κB, and MAPK in the Colonic Tissue of CAC Mice

**Figure 6:**
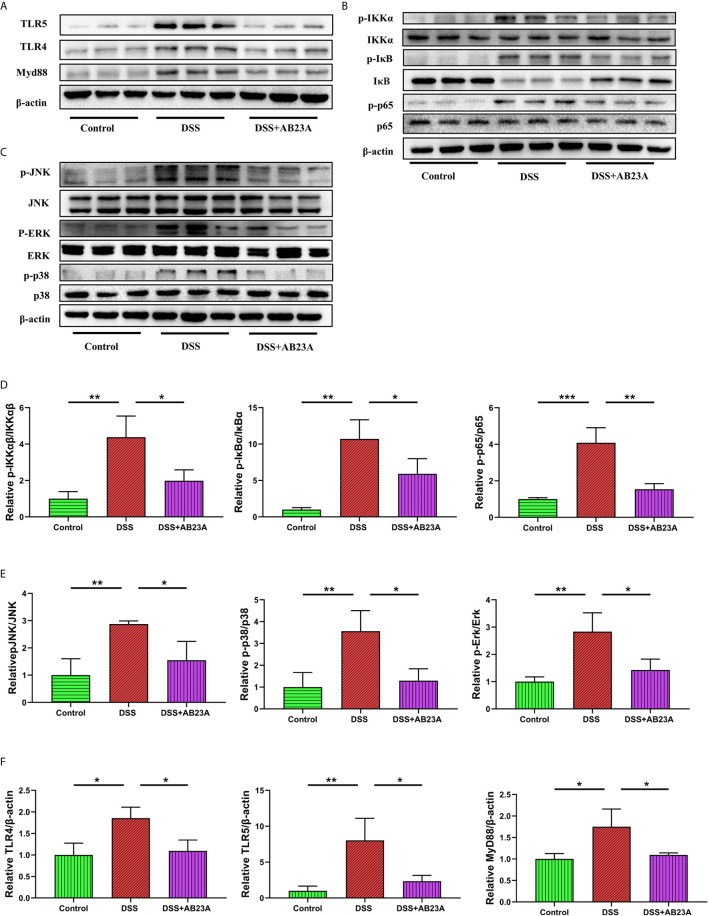
Effects of AB23A on the activation of TLRs, NF-κB and MAPK in colonic tissue of CAC mice. **(A)** Immunoblotting of TLR4, TLR5 and MyD88 in colon tissue. **(B, C)** Immunoblotting of total and phosphorylated IKKα IκBα, and p65 and total and phosphorylated JNK, ERK, and p38 in colon tissue. **(D)** p-IKKα, p-IκBα, and p-p65 protein expression relative to IKKα, IκBα, and p65. **(E)** p-JNK, p-ERK, and p-p38-MAPK protein expression relative to JNK, ERK, and p38-MAPK. **(F)** TLR4 and MyD88 protein expression relative to β-actin. Data are expressed as mean ± SD (n = 3). Differences were analyzed by one-way ANOVA followed by Tukey’s post hoc test (*P < 0.05, **P < 0.01, ***P < 0.001).

We studied TLR4, TLR5, NF-κB, and MAPK, the key efficient molecules that mediate pro-inflammatory signaling, in order to provide more insights into the modulation of inflammatory responses by AB23A. Activation of TLR4 (*P* < 0.05), TLR5 (*P* < 0.05) and MyD88 (*P* < 0.05) was inhibited by AB23A in CAC mouse colon tissue (**Figure 6A**). AB23A treatment led to consistent and remarkable reductions in the activation of IKKα (*P* < 0.05), IκBα (*P* < 0.05), and p65 (*P* < 0.01), the significant molecules needed to activate the NF-κB (**Figure 6B**). Moreover, AB23A greatly reduced the phosphorylation of p38-MAPK (*P* < 0.05), ERK (*P* < 0.05), and JNK (*P* < 0.05), the key MAPKs implicated production of pro-inflammatory mediators (**Figure 6C**). The relative protein expression results are shown in **Figures 6D–F**. Taken together, our data indicate that AB23A confers profound protective effects against CAC in mice by reducing inflammatory responses through inhibition of the activation of the NF-κB/MAPK/TLR/MyD88 signaling pathways.

### AB23A Could Maintain Intestinal Integrity and Functionality

**Figure 7:**
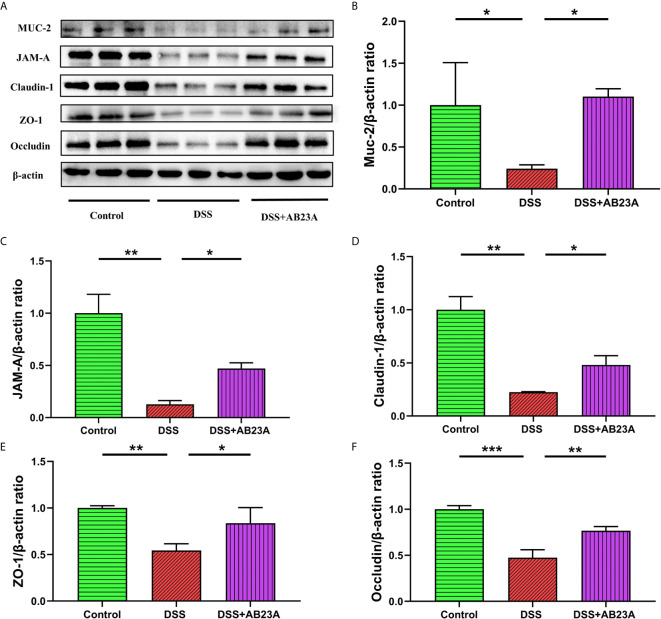
Effect of AB23A treatment on intestinal barrier functions. **(A)** Immunoblotting of mucin-2, JAM-A, claudin-1, ZO-1, and occludin. **(B–F)** Mucin-2, JAM-A, claudin-1, ZO-1, and occludin protein expression relative to β-actin. Data are expressed as mean ± SD (n = 3). Differences were analyzed by one-way ANOVA followed by Tukey’s post hoc test (*P < 0.05, **P < 0.01, ***P < 0.001).

As shown in **Figure 7A**, MUC-2 expression in CAC mice of DSS group was substantially decreased in comparison to the mice in the control group. (*P* < 0.01). However, pretreatment with AB23A significantly increased the expression of this protein (*P* < 0.05). AB23A intervention also markedly up-regulated the expression of tight junction (TJ) proteins, including junctional adhesion molecule A (JAM-A; *P* < 0.01), claudin-1 (*P* < 0.05), ZO-1 (*P*< 0.05), and occludin (*P* < 0.01), as compared with those in the DSS group (**Figures 7B–F**). Our data show that AB23A regulates the expression of TJ proteins and reduces intestinal permeability to maintain intestinal integrity and functionality.

## Discussion

**Figure 8:**
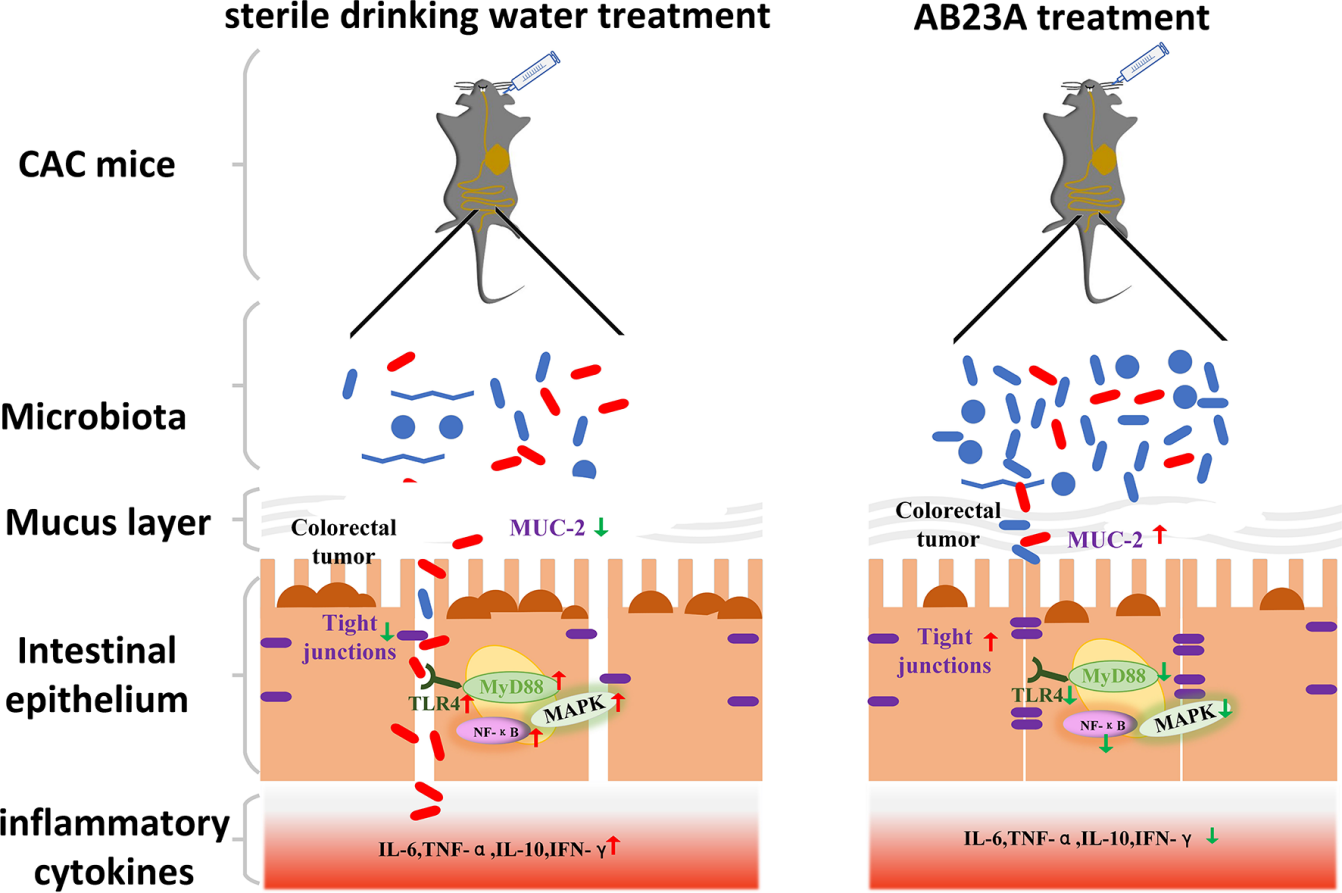
AB23A Ameliorates CAC mice model, at least partially, via modulating the composition of gut microbiota, improving the intestinal barrier and inhibiting the activation of TLR4, NF-κB, and MAPKs.

In our study, we investigated the potentially beneficial results of AB23A treatment on a CAC mouse model currently used in anti-CAC research. **Figures 8** as a summary of the results vividly shows the results of our experiment Our results showed that CAC mice’s clinical signs, such as watery urine, anal prolapse, decreased body weight, and bloody diarrhea, are greatly reduced by AB23A. These clinical symptoms are often regarded as an indicator of gut inflammation. AB23A intervention decreased inflammatory cell infiltration, atypical hyperplasia, and the number and sizes of tumors compared with those in the DSS group. The histopathological assessment has shown that AOM/DSS causes penetration and destructions of the colon epithelial barrier. Intervention with AB23A, on the other hand, significantly reduced mucosal inflammation and colon tissue damage as evidenced through notable extensions in the length of colon, low histological scores, and decreased pro-inflammatory cytokines. These results demonstrate that AB23A treatment could attenuate the symptoms of CAC mice.

A dynamic micro-ecosystem that has an important role to play in CAC prevention is the gut microbiota. Decreased diversity and richness often occur in CAC (Li C. et al., 2020). While many studies back up the idea that a low Firmicutes/Bacteroides (F/B) ratio suggests good health, the F/B value of mice in the DSS community, however, is relatively poor, according to recent research (Liu et al., 2019). Our experiments yielded results similar to these previous findings. Possible reasons for this phenomenon are individual differences in mice and environmental factors. Therefore, the F/B model used in our animal model to characterize the normal flora level of healthy mice may not be suitable. By observing the results of our experiments, the flora in the normal group of mice is characterized by low-abundance Proteobacteria and high-abundance Firmicutes, similar in the AB23A intervention group, while the flora in the DSS group of mice is characterized by extreme high-abundance Proteobacteria and low-abundance Firmicutes. Therefore, as far as characteristics of the mouse gut microbiota in our study is concerned, it is more meaningful for us to use P/F to characterize changes in the microbiota. AB23A intervention reversed the microbiota disorder and shifted it toward levels similar to those in the control group. Most of the bacteria in the DSS group were pathogenic bacteria, including Muribaculaceae, Enterobacteriaceae, Proteobacteria, *Citrobacter*, and *Klebsiella*. Probiotics such as *Alloprevotella*, *Ruminococcaceae*, *Lactobacillus*, and Bacteroidaceae (e.g., *B. vulgatus*, *B. acidifaciens*), were profoundly enriched in the AB23A group. *Alloprevotella* and Ruminococcaceae are known as anti-inflammatory factors (Meehan et al., 2014; Wei et al., 2018). *Lactobacillus* is generally used in probiotics and has been shown to prevent colon cancer in a mouse model (Friend and Shahani, 1984; Matsumoto et al., 2009). *B. acidifaciens* is known for its anti-obesity, anti-metabolic disorder, and anti-cancer properties (Huang et al., 2017). The pathogenic species of Enterobacteriaceae in CAC mice included *Klebsiella oxytoca*, *E. coli*, and *Citrobacter*, which associated with various kinds of intestinal cancers (Mullineaux-Sanders et al., 2019; Olm et al., 2019; Unterhauser et al., 2019). Recent studies have shown that members of Muribaculaceae and *Akkermansiaceae* are the key potential mucus degraders (Derrien et al., 2008; Lagkouvardos et al., 2019; Lee et al., 2019). While Akkermansiaceae can play an active role in some mouse models of metabolic diseases, recent research has discovered that in DSS-induced colitis mouse models, *Akkermansiaceae* exhibits a disorderly expansion pattern (Zhang et al., 2016). These findings imply that *Akkermansiaceae* can play different roles in the DSS-induced model and the metabolic syndrome model, or even the inverse. In this study compared with that in the AB23A group, the surge of mucus-degrading bacteria in the DSS group suggests that the intestinal barrier of CAC mice is damaged. Thus, the concerted action of pathogenic and mucin-degrading bacteria may facilitate epithelial damage and dysplasia *via* breaking down the mucus barrier caused by DSS administration (Werb, 2002). The results above indicate that AB23A might reverse gut microbiota dysbiosis by decreasing pathogenic and mucus-degrading bacteria to alleviate colitis and inhibit CAC.

Intestinal microecological disorders lead to the destruction of the intestinal barrier and changes in the composition, distribution, and metabolites of the intestinal flora, which, in turn, causes abnormal activation of signaling pathways, such as TLRs, and promotes the occurrence and development of CAC (Fukata et al., 2009, Li C. et al., 2020). The intestinal barrier consists of the mucus layer, epithelial cells, and tight junction proteins and maintains balance between the absorption of nutrients and the prevention of the invasion of toxins and bacteria in the lumen (Chelakkot et al., 2018). The mucus layer consists of mucins (e.g., mucin-2) that were released by epithelial goblet cells and the first line of defense against infectious pathogenic agents that are greatly reduced in CAC mice. (Saleiro et al., 2012). TJs, which consist of transmembrane proteins (e.g., occludin, claudin-1, and JAM-A) and intracellular membranes (e.g., ZO-1), play key roles in gut barrier permeability, adhesion between cells, and motion through the paracellular space (Chelakkot et al., 2018). Destroying TJs can lead to increased colonic permeability to harmful bacteria and toxins that cause intestinal inflammation and diarrhea that evoke the onset and growth of CAC (Saleiro et al., 2012). In this study, AB23A has substantially suppressed the declining expression of TJ proteins, (e.g., Claudin-1, ZO-1, Occludin) and mucin-2. Our research results show that the effect of AB23A in alleviating the disease state of CAC mice is largely related to enhancing the secretion of the mucus layer and protecting the tight junctions between the intestines.

Chronic inflammatory stimuli are closely linked to the development of CAC, according to epidemiologic and experimental evidence (Katoh et al., 2013). Because of increased intestinal permeability, intestinal epithelial cells and immune cells can recognize intestinal pathogenic microbes *via* Toll-like receptor (eg, TLR4, TLR5) and then activate downstream inflammation signaling pathways, including NF-κB and MAPK, to promote intestinal epithelial cell proliferation and damage repair and recruit acute inflammatory cells (Waldner and Neurath, 2015; Zou et al., 2016; Koveitypour et al., 2019; Soleimani et al., 2020). TLR activation begins activating the significant adapter molecule MyD88, which is necessary for signals from TLR pathway. In the present study, we observed that the relative abundance of inflammation-related pathogenic bacteria, such as *K. oxytoca*, *Escherichia coli*, and *Citrobacter*, significantly decreased with AB23A intervention in CAC mice. Activation of TLR4, TLR5 and MyD88 was inhibited by AB23A in CAC mouse colon tissues. AB23A decreased the phosphorylation expression of key protein of the NF-κB pathway, but increased the expression of IκBα. ERK, p38-MAPK, and JNK phosphorylation were also down-regulated by AB23A treatment. The protective activity of AB23A against CAC were discovered to be closely linked to the improvement of inflammatory status through inhibition of the TLR/NF-B/MAPK signaling pathways, according to our findings.

The development process of CAC can be divided into inflammation, dysplasia, and carcinoma (Hirano et al., 2020). Repeated inflammatory stimulation slowly develops into the abnormal proliferation of intestinal epithelial cells and even carcinogenesis. So, intestinal inflammation is one of the triggers for the development of CAC. When the epithelial barrier is damaged, invasive bacteria (usually pathogenic bacteria) can penetrate the gut barrier to trigger a pro-inflammatory response, while bacteria (usually non-pathogenic bacteria) that cannot penetrate the gut barrier stay at the face of intestinal epithelial cells and trigger a homeostasis anti-inflammatory response (Abreu, 2010). TLR is induced and activated by harmful bacteria, which causes NF-B and MAPK to be activated. In other words. The activation of NF-κB and MAPK is mainly because TLR is stimulated and activated by harmful bacteria (Xie et al., 2015; Kuai et al., 2020). In our study, AB23A intervention reversed the microbiota disorder and shifted it toward levels similar to those in the control group, which reduced stimulation of pathogenic microorganism on TLR (TLR4, TLR5). At the same time, AB23A also reduced the intestinal permeability of CAC mice. Thus, improved gut microbiota dysbiosis and maintaining intestinal integrity may be the underlying mechanisms of the preventive impact of AB23A on tumorigenesis. However, our study still has some limitations. First, the sample size of the experiment in this study is relatively small. Secondly, our study could not directly demonstrate the causal effect of microbiota change in tumorigenesis. Thus, it is essential for us to conduct in-depth and clear research in the future. Despite this some limitations, our results may still be important because AB23A induced beneficial changes in gut microbiota of CAC mice and the intestinal flora is closely related to CAC.

## Conclusion

In summary, AB23A holistically improved CAC mice clinical symptoms and the gut microenvironment by decreasing the proportion of certain pathogenic bacteria and increasing the proportion of beneficial bacteria. AB23A reduced intestinal microecological disorders and intestinal permeability in CAC mice, thus inhibiting the entry of toxins and luminal bacteria. AB23A also down-regulated proinflammatory markers by inhibiting the activation of TLR4, NF-κB, and MAPK. This study revealed that AB23A induces alterations in the gut microbiota structure, maintains intestinal integrity, and ameliorates intestinal inflammation. These synergistic effects may be an underlying cause of reduced polyp formation in CAC mice with AB23A intervention. The findings may offer insights into the role of microbiota in colon tumorigenesis. Thus, AB23A has the potential to be used to treat CAC in the future.

## Data Availability Statement

The original contributions presented in the study are publicly available. This data can be found here: https://www.ncbi.nlm.nih.gov/bioproject/PRJNA687168.

## Ethics Statement

The animal study was reviewed and approved by the Animal Ethics Committee of Fujian Medical University.

## Author Contributions

Important contributions to design and also to preparing the manuscript, WX and S-SW. Applied for funding to carry this research and design this research. WX. Designed and supervise the study, H-CZ and X-KJ. Implement specific operations for animal experiments and molecular biology experiments. S-HX, X-YL, M-QH, and M-LL. Analyzed the microbiota data, YF and H-CZ. All authors contributed to the article and approved the submitted version.

## Funding

This research was funded by National Natural Science Foundation of China (81773956, 81703692 and 81872990), the Provincial Natural Science Foundation of Fujian (2018J01870), young and middle-aged backbone Program of Fujian Provincial Health Commission (2018-ZQN-65).

## Conflict of Interest

The authors declare that the research was conducted in the absence of any commercial or financial relationships that could be construed as a potential conflict of interest.
