# Supplementary material for: Alisol B 23-Acetate Ameliorates Azoxymethane/Dextran Sodium Sulfate-Induced Male Murine Colitis-Associated Colorectal Cancer via Modulating the Composition of Gut Microbiota and Improving Intestinal Barrier
Source: Front Cell Infect Microbiol. 2021 Apr 29;11:640225. doi: 10.3389/fcimb.2021.640225 (PMC8117151; doi:10.3389/fcimb.2021.640225)

*Supplementary Information*

Supplementary Figures and Tables Legends

Table S1. The information of all antibodies used in this study

Table S2. The evaluation details of the Disease Activity Index (DAI)

Table S3. The evaluation details of the histological score

Table S4. Alpha diversity index of different groups

Table S5. The relative abundance of representative gut microbiota in Phylum, Family and Genus level.

Table S6. The development rate of dysplasia

Figure S1 Supplementary colon tumor burden pictures for Figure 1(D), (*n* = 3).

Figure S2 Supplementary colon HE pictures for Figure 2(H), (*n* = 3).

Figure S3 Supplementary immunoblots for Figure 6A(A-B).

Figure S4. Supplementary immunoblots for Figure. 6B(A-B).

Figure S5 Supplementary immunoblots for Figure. 6C (A-B).

Figure S6 Supplementary immunoblots for Figure 7A(A-B).

| Antibodies | Manufactor | Source | Identifier | Dilution |
| --- | --- | --- | --- | --- |
| Anti-JNK | [Abcam](http://www.baidu.com/link?url=0nuLlStyeteufKxaB-8UHkGN_wBJV7ncOLQXF6ptmN4HM5HsxZYnh2vr7n6QUoeRFkL77dLp-yaVFcuFF3cD7ed6zq8YoadM9Hx6SHw5HOq) | Rabbit | ab179461 | 1:1000 |
| Anti-p-JNK | [Abcam](http://www.baidu.com/link?url=0nuLlStyeteufKxaB-8UHkGN_wBJV7ncOLQXF6ptmN4HM5HsxZYnh2vr7n6QUoeRFkL77dLp-yaVFcuFF3cD7ed6zq8YoadM9Hx6SHw5HOq) | Rabbit | ab124956 | 1:1000 |
| Anti-ERK | Cell Signaling Technology | Rabbit | 4695S | 1:1000 |
| Anti-p-ERK | Cell Signaling Technology | Rabbit | 4370T | 1:1000 |
| Anti-p38 | Cell Signaling Technology | Rabbit | 3010s | 1:1000 |
| Anti-p-p38 | Cell Signaling Technology | Rabbit | 4511T | 1:1000 |
| Anti-NF-κB(p-65) | Proteintech | Rabbit | 10745-1-AP | 1:1000 |
| Anti-p-NF-κB(p-p-65) | [Cell Signaling Technology](http://www.baidu.com/link?url=0nuLlStyeteufKxaB-8UHkGN_wBJV7ncOLQXF6ptmN4HM5HsxZYnh2vr7n6QUoeRFkL77dLp-yaVFcuFF3cD7ed6zq8YoadM9Hx6SHw5HOq) | Rabbit | 3033T | 1:1000 |
| Anti-IκBα | [Cell Signaling Technology](http://www.baidu.com/link?url=0nuLlStyeteufKxaB-8UHkGN_wBJV7ncOLQXF6ptmN4HM5HsxZYnh2vr7n6QUoeRFkL77dLp-yaVFcuFF3cD7ed6zq8YoadM9Hx6SHw5HOq) | Mouse | 4814S | 1:1000 |
| Anti-p-IκBα | [Cell Signaling Technology](http://www.baidu.com/link?url=0nuLlStyeteufKxaB-8UHkGN_wBJV7ncOLQXF6ptmN4HM5HsxZYnh2vr7n6QUoeRFkL77dLp-yaVFcuFF3cD7ed6zq8YoadM9Hx6SHw5HOq) | Rabbit | 2859S | 1:1000 |
| Anti-IKKα/β | [Cell Signaling Technology](http://www.baidu.com/link?url=0nuLlStyeteufKxaB-8UHkGN_wBJV7ncOLQXF6ptmN4HM5HsxZYnh2vr7n6QUoeRFkL77dLp-yaVFcuFF3cD7ed6zq8YoadM9Hx6SHw5HOq) | Mouse | 11930S | 1:1000 |
| Anti-p-IKKα/β | [Cell Signaling Technology](http://www.baidu.com/link?url=0nuLlStyeteufKxaB-8UHkGN_wBJV7ncOLQXF6ptmN4HM5HsxZYnh2vr7n6QUoeRFkL77dLp-yaVFcuFF3cD7ed6zq8YoadM9Hx6SHw5HOq) | Rabbit | 2697T | 1:1000 |
| Anti-TLR4 | Proteintech | Mouse | 66350-1-Ig | 1:1000 |
| Ant-MyD88 | Proteintech | Rabbit | 23230-1-AP | 1:1000 |
| Anti-ZO-1 | Proteintech | Rabbit | 13409-1-AP | 1:1000 |
| Anti-Occludin | Proteintech | Rabbit | 21773-1-AP | 1:1000 |
| Ant-Claudin-1 | Proteintech | Rabbit | 13050-1-AP | 1:1000 |
| Ant-Mucin-2 | Cell Signaling Technology | Mouse | #88686 | 1:1000 |
| Ant-JAM-A  β-actin | Abcam  Proteintech | Rabbit  Mouse | ab52647  66009-1-Ig | 1:1000  1:10000 |

Supplementary Tables

Table S1

Table S2

| DAI Score | | | |
| --- | --- | --- | --- |
| Score | Weight loss | Stool consistency | Rectal bleeding |
| 0 | None | Normal | Normal |
| 1 | 1-5% | — | — |
| 2 | 5-10% | Loose stools |  |
| 3 | 10-20% | — | — |
| 4 | >20% | Diarrhea | Gross bleeding |

Table S3

| Histological score | | |
| --- | --- | --- |
| Mucosal epithelium | 0 | No mucosa inflammation |
|  | 1 | Loss of <5% of the epithelial surface |
|  | 2 | Loss of 5-10% of the epithelial surface |
|  | 3 | Loss of >10% of the epithelial surface |
| Integrity of crypts | 0 | Intact crypts |
|  | 1 | Loss of <10% crypts |
|  | 2 | Loss of 10-20% crypts |
|  | 3 | Loss of >20% of crypts |
| Cell infiltrate and edema | 0 | None |
|  | 1 | Mild |
|  | 2 | moderate |
|  | 3 | severe |
| Goblet cells depletion | 0 | Absent |
|  | 1 | Present |

Table S4

| Group | chao1 | observed_species | shannon | simpson | ACE | PD_whole_tree |
| --- | --- | --- | --- | --- | --- | --- |
| Control | 431.1±82.72 | 394.8±81.99 | 5.243±0.7524** | 0.9188±0.0651** | 0.9986±0.0005164 | 27.95±6.111 |
| DSS | 267.5±96.55## | 235.2±84.23## | 2.845±0.3725 | 0.7309±0.04363 | 0.9987±0.000483## | 26.62±6.876 |
| DSS+AB23A | 343.1±95.76# | 307.5±92.01# | 3.999±0.7344** | 0.8049±0.1099** | 0.9985±0.000527# | 23.34±7.173 |

Data are expressed with Mean±SD (*n* = 10). (*vs*. DSS: *^*^P* < 0.05, *^**^P* < 0.01; *vs*. Control: *^#^P* < 0.05, *^##^P* < 0.01).

Table S5

| Species | Taxonomy | Control | DSS | DSS+AB23A |
| --- | --- | --- | --- | --- |
| Bacteroidetes | Phylum | 0.40±0.20 | 0.33±0.12 | 0.62±0.13^##^ |
| *Firmicutes* | Phylum | 0.46±0.14^**^ | 0.04±0.03 | 0.31±0.12^##^ |
| *Proteobacteria* | Phylum | 0.062±0.05^**^ | 0.61±0.12 | 0.03±0.03^##^ |
| *Enterobacteriaceae* | Family | 1.37±2.17^**^ | 55.99±10.87 | 0.34±0.22^##^ |
| *Prevotellaceae* | Family | 5.94±7.41^*^ | 0.20±0.18 | 2.88±2.33^##^ |
| *Ruminococcaceae* | Family | 21.91±12.43^**^ | 0.48±0.49 | 22.51±13.50^##^ |
| *Muribaculaceae* | Family | 7.87±7.58^**^ | 28.70±11.87 | 1.08±1.05^##^ |
| *Bacteroidaceae* | Family | 14.52±10.68^**^ | 0.35±0.22 | 50.56±11.91^##^ |
| *Rikenellaceae* | Family | 4.82±4.87^**^ | 0.11±0.09 | 5.89±3.26^##^ |
| *Akkermansiaceae* | Family | 0.02±0.02^*^ | 0.13±0.16 | 0.01±0.01^#^ |
| *Bacteroides* | Genus | 0.14±0.11^**^ | 0.00±0.00 | 0.51±0.12^##^ |
| *Lactobacillus* | Genus | 0.078±0.09^*^ | 0.02±0.01 | 0.02±0.01 |
| *Alistipes* | Genus | 0.05±0.04^*^ | 0.00±0.00 | 0.06±0.03^##^ |
| *Klebsiella* | Genus | 0.00±0.00^**^ | 0.41±0.07 | 0.00±0.00^##^ |
| *Unidentified_Enterobacteriaceae* | Genus | 0.01±0.01^*^ | 0.02±0.01 | 0.00±0.00^##^ |
| *Unidentified_Ruminococcaceae* | Genus | 0.12±0.08^**^ | 0.00±0.00 | 0.14±0.11^##^ |
| *Alloprevotella* | Genus | 0.06±0.07^*^ | 0.00±0.00 | 0.01±0.01^#^ |
| *Citrobacter* | Genus | 0.00±0.00^**^ | 0.01±0.02 | 0.00±0.00^##^ |
| *Pantoea* | Genus | 0.00±0.00^**^ | 0.01±0.00** | 0.00±0.00^##^ |

Data are expressed with Mean ± SD *(n* = 10). (*vs*. DSS: *^*^P* < 0.05, *^**^P* < 0.01, *vs*. DSS, *^#^P* < 0.05, *^##^P* < 0.01).

Table S6.

| Group | number of samples | number of dysplasia | the development rate of dysplasia (%) |
| --- | --- | --- | --- |
| Control | 10 | 0 | 0 |
| DSS | 10 | 10 | 100 |
| DSS+AB23A | 10 | 10 | 100 |

Supplementary Figures

Figure S1


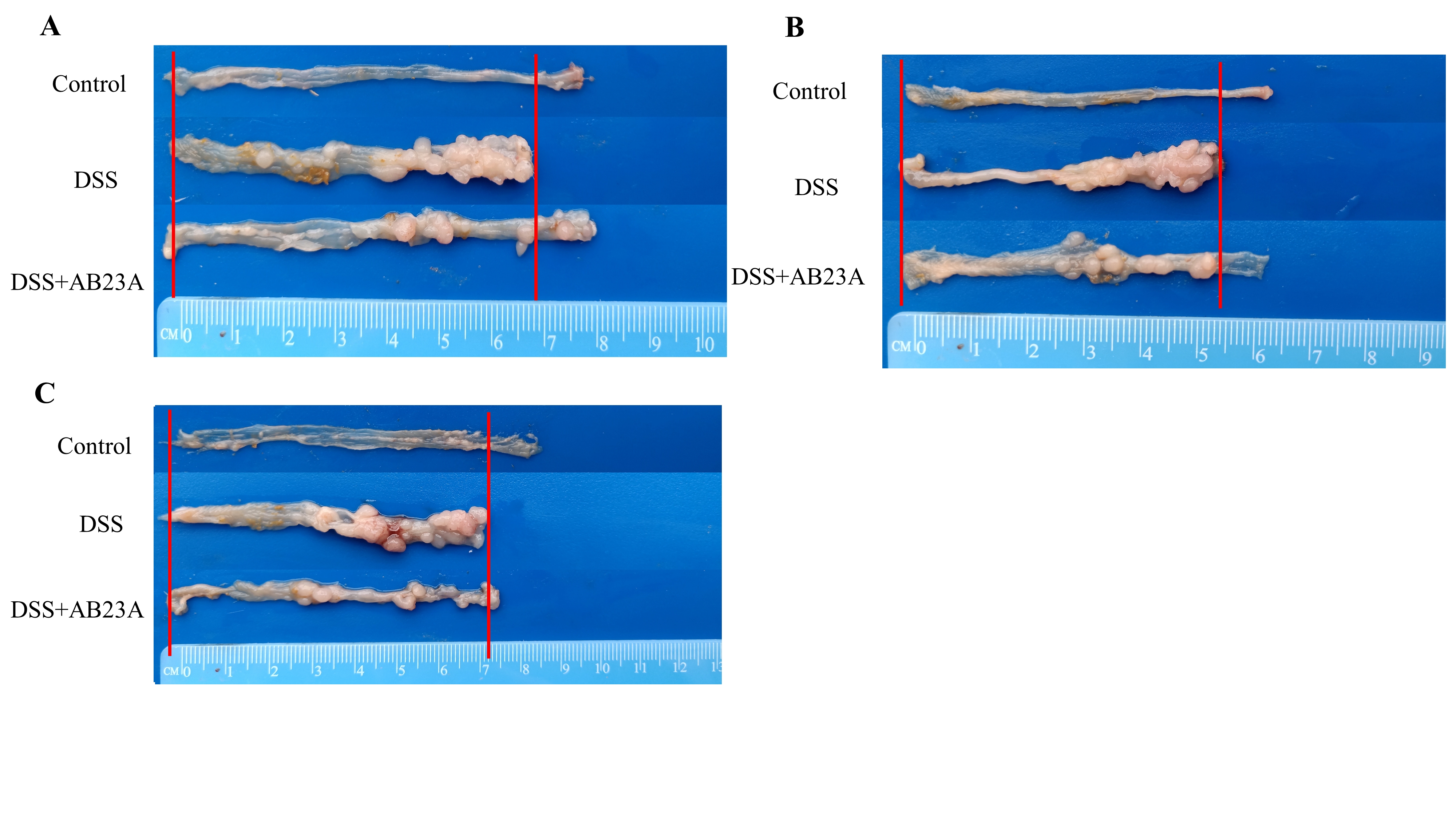


Figure S2


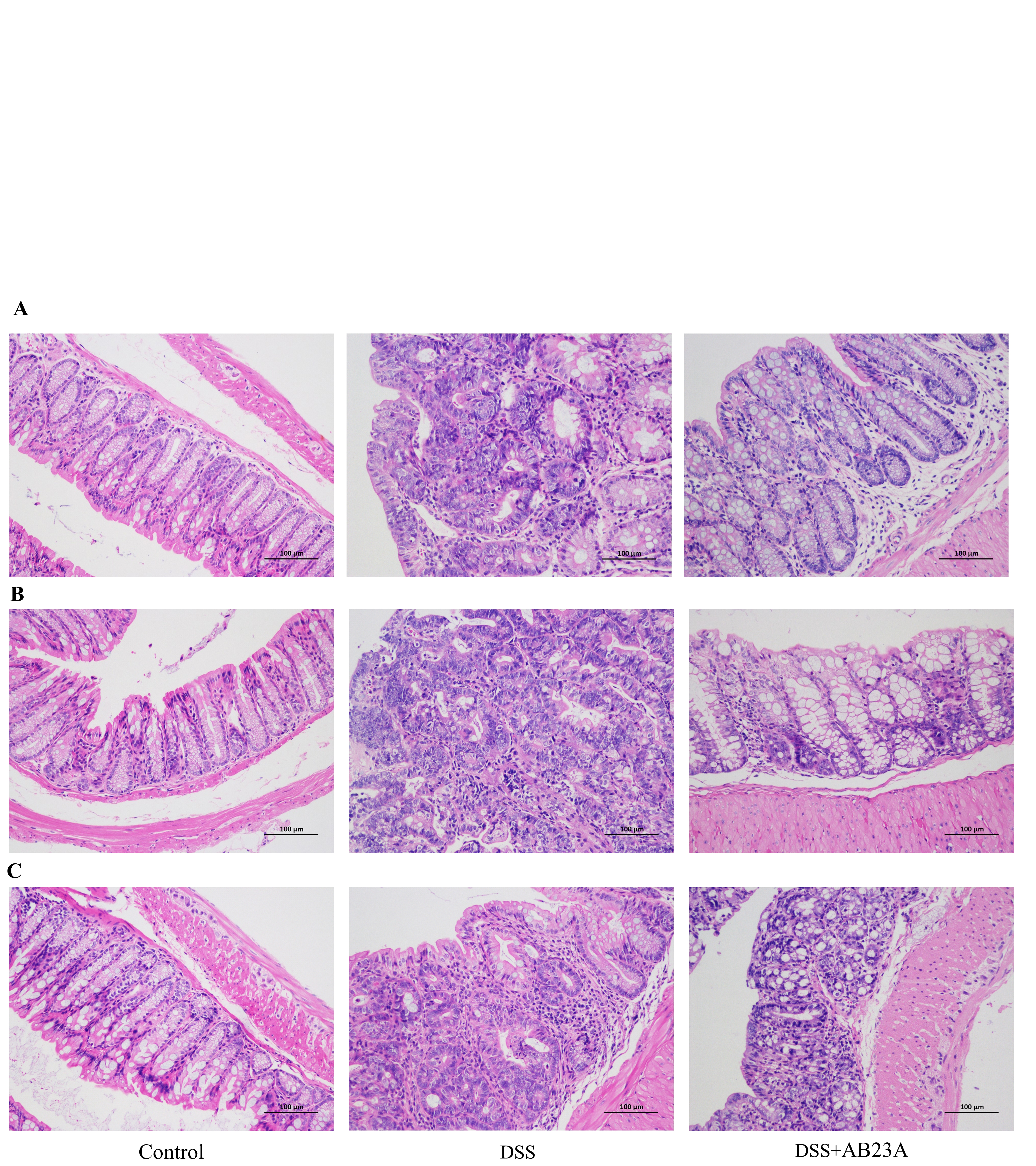


Figure S3


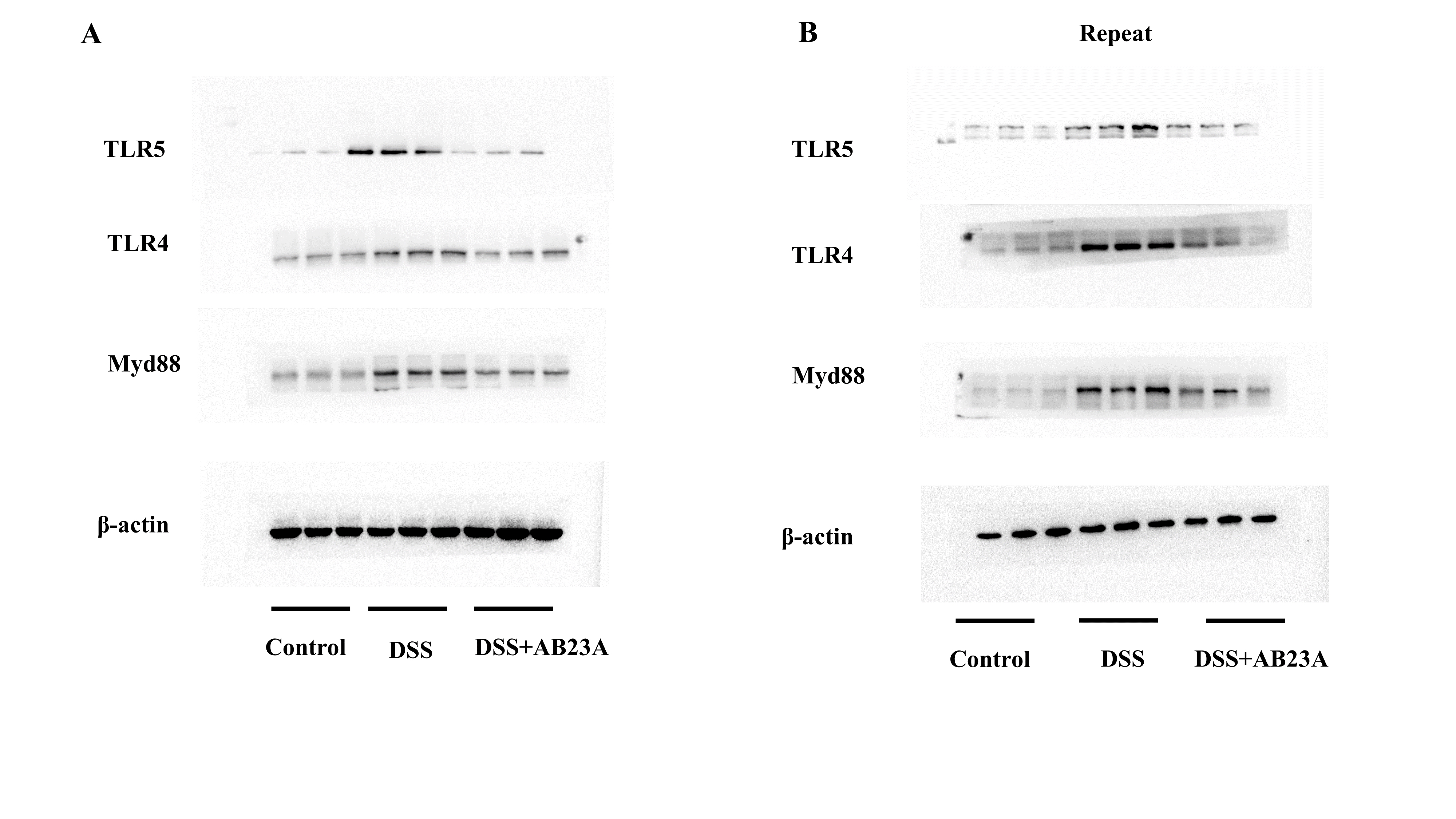


Figure S4


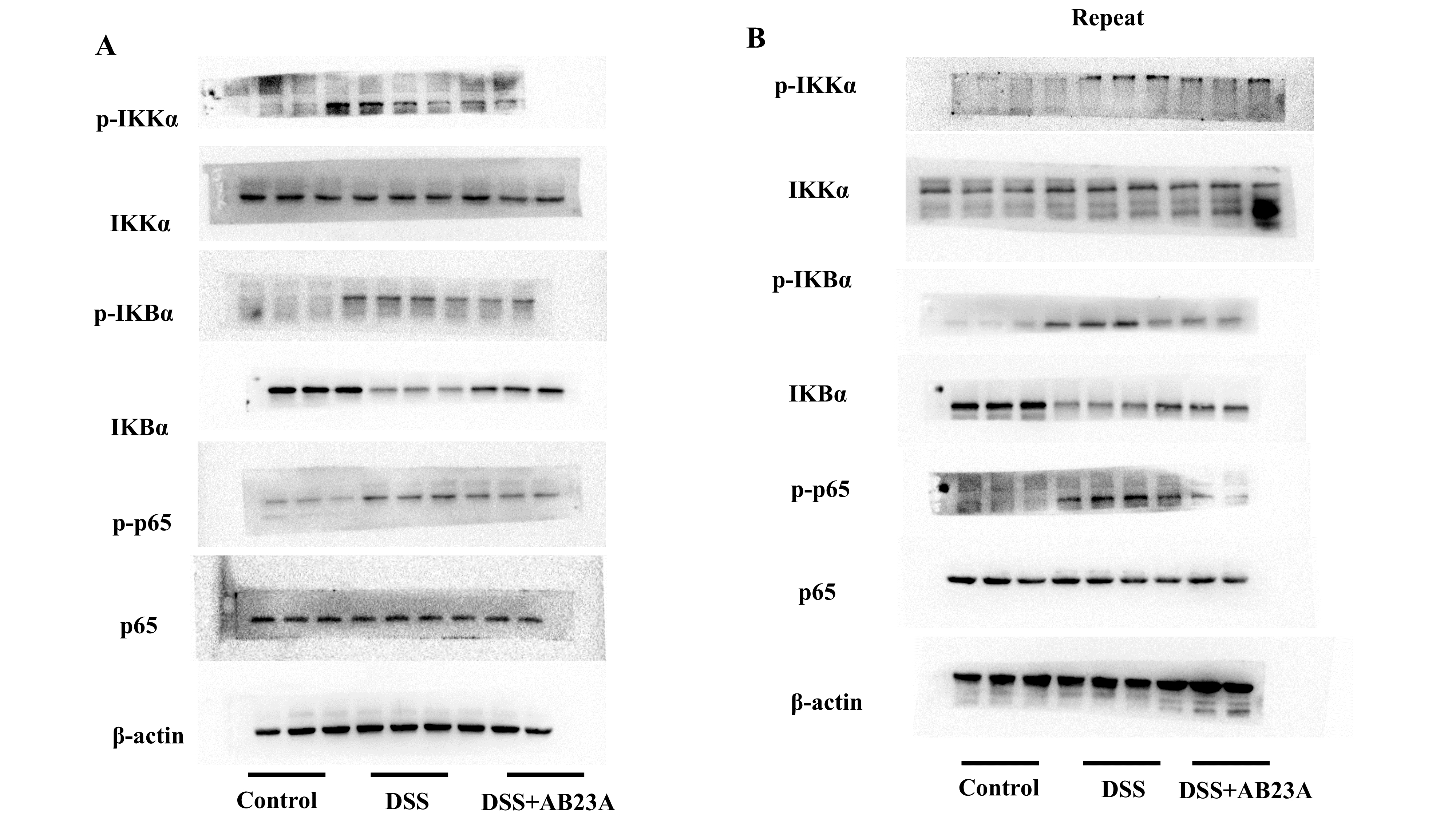


Figure S5


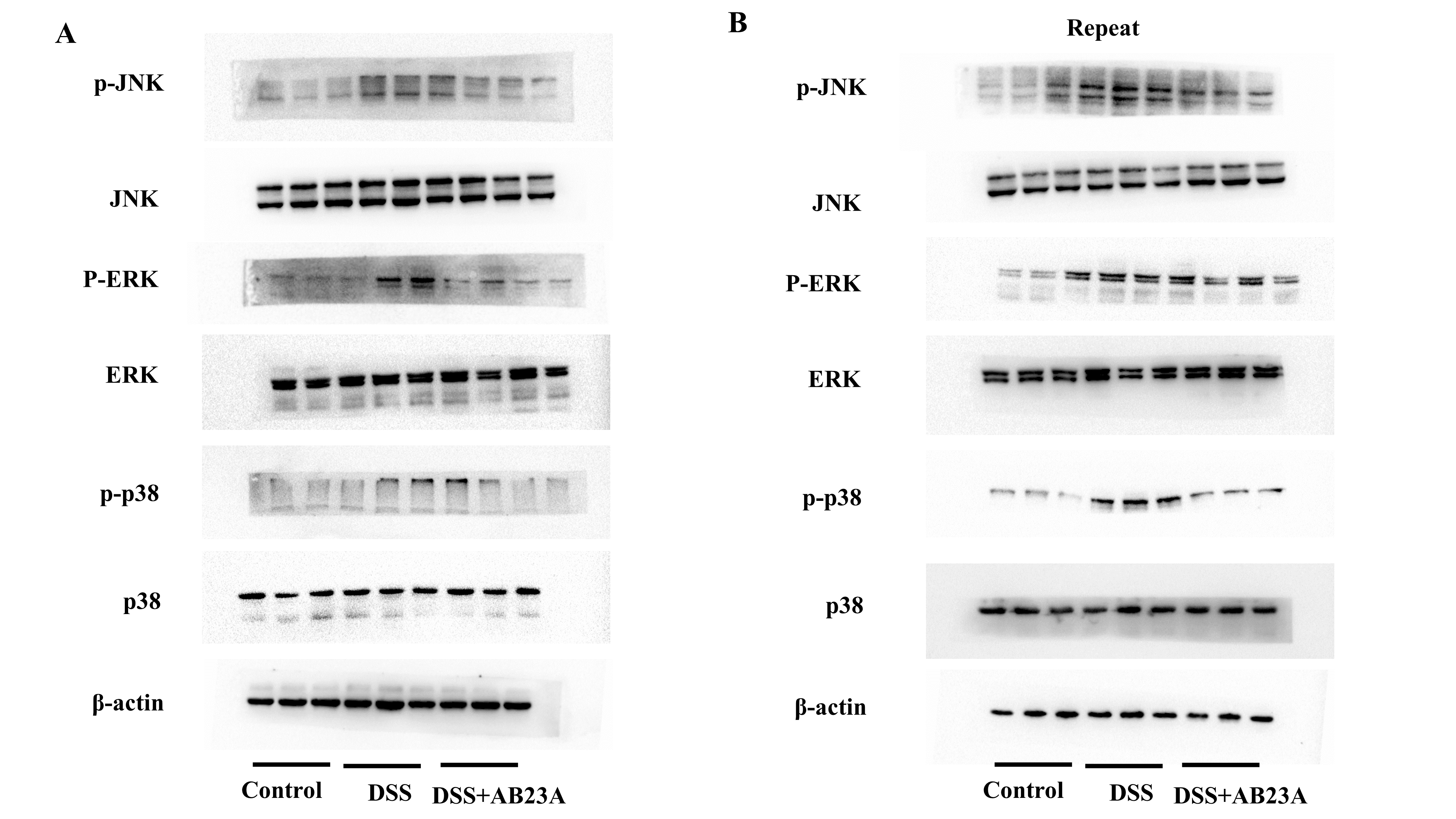


Figure S6


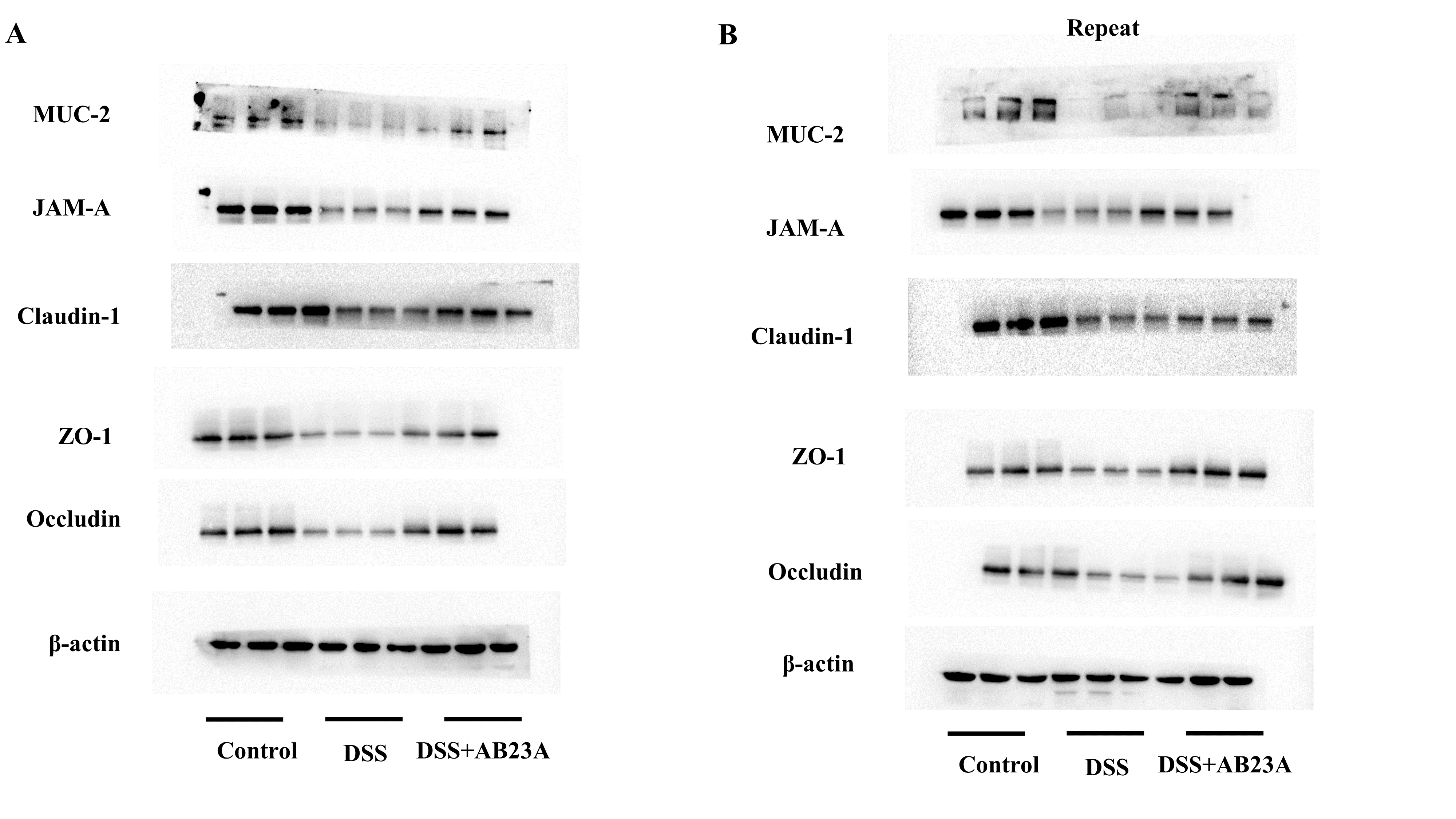

Supplement: Supplementary file 1 [file DataSheet_1.docx]
